# Molecular Effects of *Xylella fastidiosa* and Drought Combined Stress in Olive Trees

**DOI:** 10.3390/plants8110437

**Published:** 2019-10-23

**Authors:** Mariarosaria De Pascali, Marzia Vergine, Erika Sabella, Alessio Aprile, Eliana Nutricati, Francesca Nicolì, Ilaria Buja, Carmine Negro, Antonio Miceli, Patrizia Rampino, Luigi De Bellis, Andrea Luvisi

**Affiliations:** 1 Department of Biological and Environmental Sciences and Technologies, University of Salento, 73100 Lecce, Italy; mariarosaria.depascali@unisalento.it (M.D.P.); erika.sabella@unisalento.it (E.S.); alessio.aprile@unisalento.it (A.A.); eliana.nutricati@unisalento.it (E.N.); francesca.nicoli@unisalento.it (F.N.); carmine.negro@unisalento.it (C.N.); antonio.miceli@unisalento.it (A.M.); patrizia.rampino@unisalento.it (P.R.); luigi.debellis@unisalento.it (L.D.B.); andrea.luvisi@unisalento.it (A.L.); 2 Department of Mathematics and Physics “Ennio De Giorgi”, University of Salento, 73100 Lecce, Italy; ilaria.buja@unisalento.it

**Keywords:** abiotic-biotic stress, combined stress, water deficit, plant disease, pathogen tolerance

## Abstract

Due to global climate change, complex combinations of stresses are expected to occur, among which the interaction between pathogens and drought stress may have a significant effect on growth and yield. In this study, the *Xylella fastidiosa* (*Xf*)-resistant *cultivar* Leccino and the susceptible one Cellina di Nardò were subjected to (a) individual drought stress, (b) *Xf* infection and (c) combination of both stress conditions. Here we report the physiological response to stresses in water content in leaves and the modulation in the expression level of seven genes responsive to plant water status and pathogen infection. In *Xf*-resistant plants, higher expression levels are reported for genes belonging to ROS-scavenging systems and for genes involved in pathogen stress (pathogenesis-related, *PR*, and leucine-rich repeat genes, *LRR-RLK*). However, *PR* and *LRR-RLK* were not further induced by water deficit. Interestingly, the genes related to drought response (aquaporin, *PIP2.1*, dehydration responsive element binding, *DREB*, and dehydrin, *DHN*), which induction was higher in Cellina di Nardò compared to Leccino during drought stress, was poorly induced in *Xf*-susceptible plants when *Xf* occur. Conversely, *DHN* was induced by *Xf* presence in Leccino. These results were consistent with observations on water content. Indeed, response was similar in Leccino regardless kind of stress or combination, whereas a strong reduction was observed in *Xf*-susceptible plants infected by *Xf* or in presence of combined stresses. Thus, the reported findings indicate that resistance of Leccino to *Xf* could be linked to its lower resistance to water stress, probably leading to the activation of alternative defense pathways that support the plant in *Xf* response.

## 1. Introduction

In field conditions, plants are exposed to different environmental stresses. The molecular mechanisms underlying stress tolerance have been intensely studied [1,2,3] and the molecular mechanisms of tolerance in response to individual stresses have been explored [4,5,6,7]. However, studies on their combined effect are less common, despite being strongly associated and having a severe impact on growth and productivity [8,9,10]. When plants are exposed to different stress combinations, a variety of interacting signal transduction pathways are induced [11]. The interaction between these pathways can either be neutral, additive, synergistic or may lead to novel unpredictable responses [12,13]. In most cases, plant responses to combined stresses deviate from responses to the individual stresses [14,15]. Among different stress combinations that occur in field conditions, the combination of drought and pathogen stress is a relevant topic, since drought stress can positively or negatively affect pathogen infection [16]. In some studies, drought stress increases the susceptibility to bacterial pathogens [17], conversely, in other studies, drought stress has also been shown to enhance the tolerance toward bacteria pathogens [18,19]. Host resistance and water deficit stress tolerance are controlled by complex mechanisms and share several basal plant defense strategies. The stresses induce the modulation of the enzymatic antioxidant system, involving many enzymes, such as superoxide dismutase (SOD), catalase (CAT), and peroxidases (POX) including ascorbate peroxidase (APX), providing a highly efficient system for maintaining reactive oxygen species (ROS) homeostasis in various sites of plant cell [20]. Furthermore, numerous regulatory and/or protective proteins involved in stresses response such as aquaporins (AQPs), dehydration responsive element binding (DREB), and dehydrin proteins (DHNs) confer outstanding ability to resist drought [21], while the most commonly induced proteins during plant pathogens defense mechanisms are pathogenesis-related (PR) protein and leucine-rich repeat receptor like protein kinase (LRR-RLK).

At the end of 2013 a quarantine plant pathogen was recognized in Southern Italy (Salento peninsula, located in the Apulia region), associated to a previously unknown disease on olive trees (*Olea europaea* L.) which cause a leaf scorch, a rapid decline and the death of trees (the so-called “Olive Quick Decline Syndrome”, OQDS) [22]. These symptoms are particularly severe on plants of the *cv* Cellina di Nardò (hereafter Cellina), whereas *cv* Leccino seems weakly attacked [23,24]. The evidence and the following research studies indicated a connection with the bacterium *Xylella fastidiosa* subsp. *pauca* ‘De Donno’ (*Xf*) [25]. The bacterium exists as an endophytic commensal and spreads from the site of infection to colonize the xylem, whereas the subsequent vessel occlusion [26] induces plants to drought stress conditions and symptom development, which may be worsened by abiotic stress (e.g., the leaf scorch, which characterize the symptom of OQDS before wilting of branch, reduce the performance of photosynthetic apparatus, which may be further affected by water deficit in soil [27]. Furthermore, the studies about the potential distribution of *Xf* in current and future climate conditions forecast the presence of the bacterium in many regions of the Mediterranean area [28,29], one the most vulnerable area in the world to the impacts of global warming [30]. In this contest, the combined effect of a xylem-affecting pathogen and drought-stress highlights new challenges for plant management and protection in territories threatened by *Xf*. Thus, the knowledge of drought-related transcriptional mechanisms involved in contrasting a drought-inducer pathogen in water-deficit conditions should drive the search for resistant plants, which represent the most promising strategy to hinder the disease.

In this paper, we investigate the host’s transcriptional responses to *Xf* infection and drought in two *cvs*, Cellina (*Xf*-susceptible) and Leccino (*Xf*-resistant) under individual stress (drought or pathogen stress) and combined stress in field conditions, evaluating the change in relative water content and expression of genes coding for enzymes related to ROS scavenging activity, water deficit and pathogen stress response.

## 2. Results

### 2.1. Estimation of Water and Proline Content and Enzyme Assays

**Figure 1:**
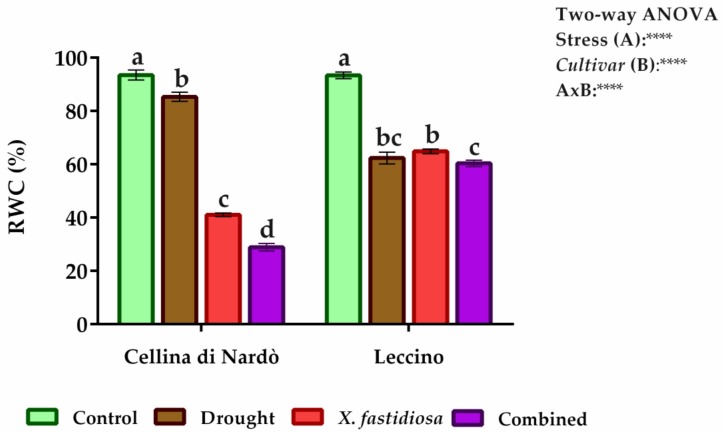
Relative water content (RWC) determined on Cellina di Nardò and Leccino fully expanded leaves subjected to individual and combined stresses (drought and *Xylella fastidiosa*). Top right Two-way ANOVA results were reported. Different letters correspond to statistically different means.

The physiological characterization of the two *cvs* analyzed in their response to drought, pathogen, and combined stresses were performed by measurement of relative water content (RWC) (Figure 1). The RWCs of control plants were not significantly different among the analyzed plants (RWC ~94% for both *cvs*). In samples subjected to drought stress, RWCs measured were lower in Leccino (with a value of about 63%) compared to Cellina (with a higher value of about 85%), indicating a better water status under absence of irrigation (*p* < 0.0001). This result is consistent with a commonly recognized tolerance to water stress of Cellina compared to Leccino. Conversely, in plants subjected to pathogen stress, the decrease of RWC value was more drastic in Cellina compared to Leccino. This behavior could be linked to the different impacts on vascular system caused by the pathogen in *cv Xf*-susceptible (C_t_ 24–27) compared to resistant one (C_t_ 28–30). However, our findings indicate that the RWC in *Xf*-positive Leccino samples was similar to that observed in drought stress (~65%), indicating a mild effect of the pathogen on water content. Conversely, a sharp decline was observed in Cellina samples (~41%).

Moreover, in the drought/pathogen combined stress, the *cv* Cellina registered a further significant reduction in RWC value (reaching about 29% of RWC), while Leccino samples showed the similar performance compared to individual stresses (~60%), confirming how the pathogen presence is not more suffered than drought by this *cv*.

**Figure 2:**
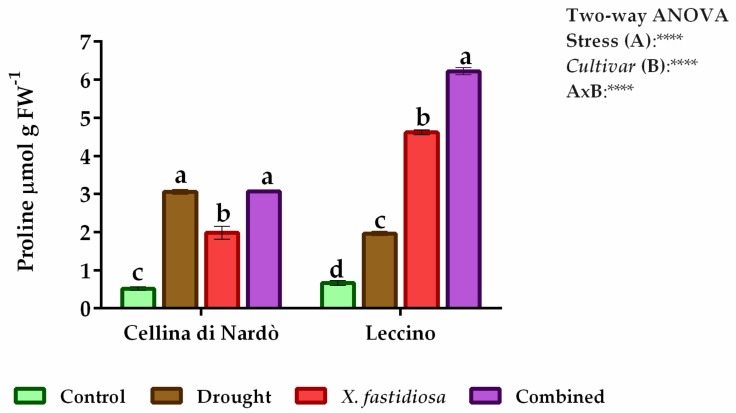
Proline content (µmol g FW^−1^) determined on Cellina di Nardò and Leccino leaves subjected to individual and combined stresses (drought and *Xylella fastidiosa*). Small letter compares the mean of five repetitions (Tukey HSD post hoc test *p* ≤ 0.05). Top right Two-way ANOVA results were reported.

Cellina and Leccino *cvs* have shown a low constitutive content of free proline (Figure 2). On the contrary, the proline content in the leaves of both *cvs* was significantly higher in the stressed plants.

In particular, the Cellina samples subjected to drought stress showed significantly more proline content (3.05 µmol g FW¯¹) than Leccino (1.96 µmol g FW¯¹). Moreover, in plants subjected to pathogen stress, the proline content increased in Leccino (4.61 µmol g FW¯¹) and decreased in Cellina (1.98 µmol g FW¯¹) compared to drought stress.

In drought/pathogen combined stress the proline content in Cellina (3.06 µmol g FW¯¹) achieve the same level registered in drought stress, whereas in Leccino (6.22 µmol g FW¯¹) have been observed an additive effect of the two individual stresses.

Furthermore, the activities of antioxidant enzymes showed a significant increase in values in both *cultivars* under individual and combined stresses compared with control plants (Table 1). Under all stress conditions considered, the enzyme activity of APX, CAT, and SOD showed a higher level in Leccino compared to Cellina. In particular, the highest enzyme activity was measured in Leccino for APX (12.30 EU mg¯¹) and SOD (28.49 EU mg¯¹) enzymes under combined stresses, while the higher value for CAT activity (15.24 EU mg¯¹) was reported under sole pathogen stress.

### 2.2. Gene Expression Analysis under Individual and Combined Stresses

**Figure 3:**
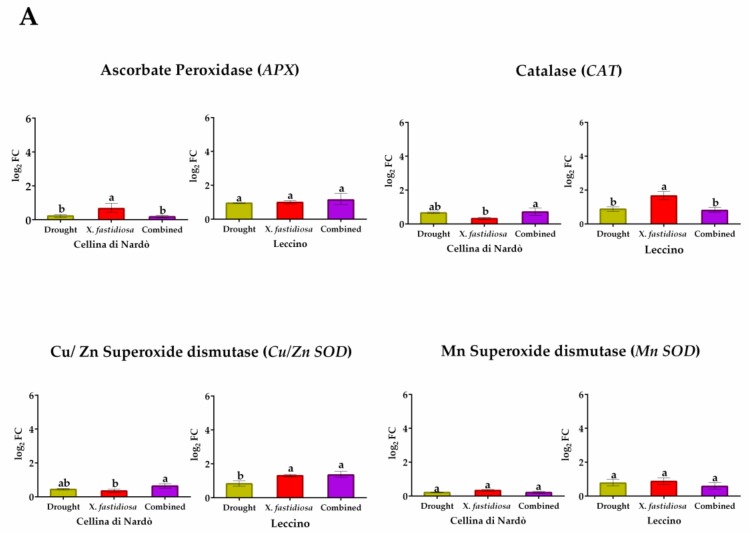

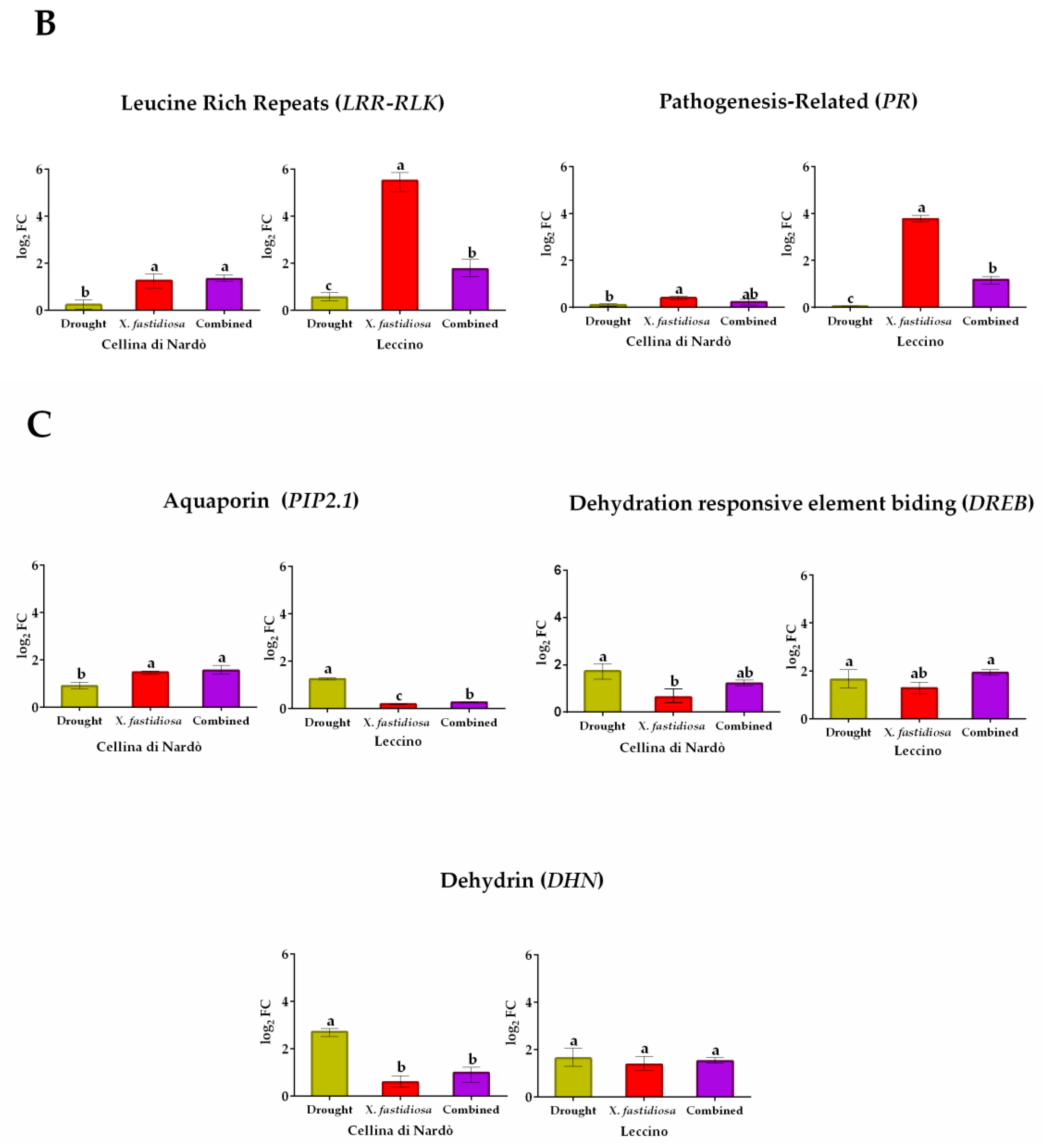
Expression analysis of stress responsive gene in leaves of Cellina di Nardò and Leccino *cultivars* subjected to stresses: drought, pathogen *Xylella fastidiosa* and combination of both, expressed as log_2_ fold change (log_2_FC). **A.** genes related to oxidative stress: superoxide dismutase (*Cu/Zn SOD* and *MnSOD*), catalase (*CAT*), ascorbate peroxidase (*APX*), **B.** genes related to pathogen stress: leucine rich repeats- receptor like kinase (*LRR-RLK*) and pathogenesis-related protein 1-like (*PR*). **C.** genes related to drought responses: aquaporin (*PIP2.1*), dehydration responsive element binding (*DREB*) and dehydrin (*DHN*). Statistical analysis was carried out through one-way ANOVA with Tukey-HSD post hoc test. Different letters correspond to statistically different means.

In Cellina (Figure 3), genes related to ROS-scavenging systems were weakly modulated by single or combined stresses. The drought stress causes a limited effect on the expression of the four selected genes, with an increase in *CAT* and *Cu/Zn SOD* expression (respectively of ~0.65 and 0.43 log_2_ FC value). Conversely, the pathogen causes an increase in the expression level of *APX*, while other genes are just slightly overexpressed. Furthermore, the addition of drought stress seems to hide the *Xf* presence. Regarding genes related to pathogen responses, both are unaffected by drought. Conversely, higher expression was observed for both *LRR-RLK* and *PR* when the sole pathogen was present (respectively 1.26 and 0.40 log_2_ FC value). The response was quite unmodified by adding water stress. About the gene related to water stress, aquaporin (*PIP2.1*) increased the transcript level in all stress conditions. *DREB* and *DHN* strongly respond to drought (respectively 1.73 and 2.70 log_2_ FC value), whereas *Xf* causes a very much lower expression in both genes, which was unmodified by a combination of water stress.

In Leccino (Figure 3), genes related to ROS-scavenging systems are affected by single or combined stresses. The drought stress causes a similar but significant effect on all selected genes. The pathogen causes significantly higher expression in *CAT* and *Cu/Zn SOD* expression (respectively of ~1.65 and 1.30 log_2_ FC value), while the addition of drought stress did not change the expression for *CAT* gene, in which the effect of pathogen seems hidden. With regard to genes related to pathogen responses, both are unaffected by drought, but we observed a predictable very high expression of *LRR-RLK* and *PR* (respectively 5.51 and 3.76 log_2_ FC value) in *Xf*-infected plants. However, the combined presence of water stress strongly reduces the expression of both genes. In relation to the gene related to water stress, *PIP2.1* showed low expression levels in all stress conditions considered, while *DREB* and *DHN* respond significantly to drought (1.73 and 1.63 log_2_ FC value), and the gene was also induced regardless of *Xf* presence or combined stresses.

**Figure 4:**
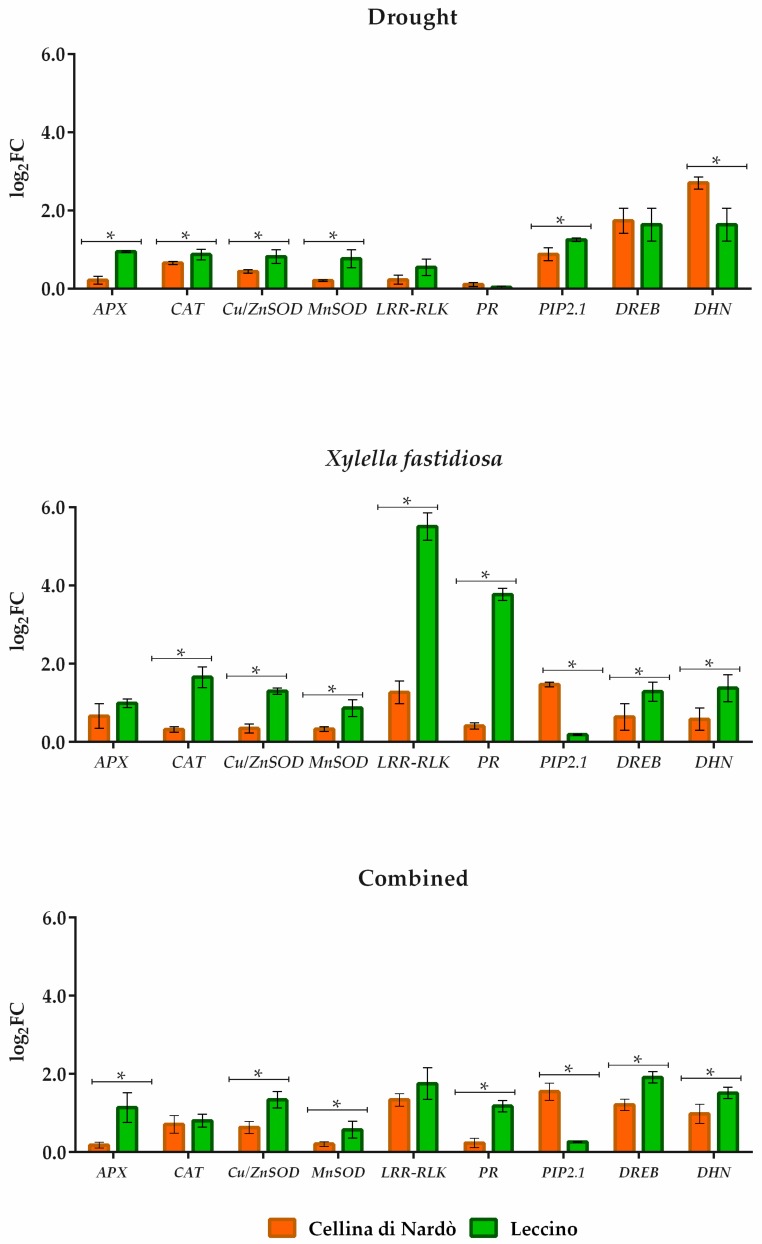
Comparison of Cellina di Nardò and Leccino *cultivars* in gene expression level subjected to drought, pathogen *Xylella fastidiosa* and combination of both stresses expressed as log_2_ fold change (log_2_FC). The genes related to oxidative stress are: Superoxide dismutase (*Cu/Zn SOD* and *MnSOD*), Catalase (*CAT*), Ascorbate Peroxidase (*APX*), the genes related to pathogen stress are: Leucine Rich Repeats (*LRR-RLK*) and Pathogenesis-Related (*PR*) and the gene related to drought responses are: Dehydrin (*DHN*). The statistical analysis was carried out using multiple t-tests (FDR = 5%).

The profile expression of genes was different among *cvs* (Figure 4). Comparing Cellina and Leccino in gene expression level subjected to drought, we observed that genes related to ROS-scavenging systems are higher expressed in *Xf*-resistant plants compared to *Xf*-susceptible ones. As predictable, any differences were observed on genes related to pathogen response, whereas the expression of *DHN* was significantly higher in the drought-tolerant Cellina compared to Leccino.

The presence of *Xf* as a stress factor underlines a quite completely different profile of gene expression among the two *cvs*. All genes related to ROS-scavenging systems but *APX* are higher expressed in *Xf*-resistant plants compared to *Xf*-susceptible ones, while the pathogen induces significantly higher expression of both genes (*LRR-RLK* and *PR*) in Leccino compared to *Xf*-susceptible plants. Interestingly, the genes involved in drought stress (*DREB* and *DHN*), are more expressed in *Xf*-resistant but drought-susceptible plants (Leccino) than in *Xf*-susceptible but drought-tolerant ones (Cellina). Conversely, *PIP.2.1* is more expressed in Cellina *Xf*-susceptible but drought resistant plants.

The addition of drought stress to the presence of the pathogen confirm a three out of four higher expressions of genes related to ROS-scavenging systems but *CAT*, which is similarly expressed in both *cultivars*, suggesting an additive effect of both stresses on this group of genes. About genes related to pathogen responses, the drought seems to cause a synergic effect on *LRR-RLK* in Cellina, which expression level becomes comparable to that observed in Leccino. The different behavior among *cvs* of *DHN* observed in pathogen single stress was confirmed when drought stress was added.

## 3. Discussion

Plants are constantly subjected to both abiotic and biotic stresses and the responses to these stresses are complex and involve numerous physiological, molecular, and cellular adaptations that cause the change in the crop yield and quality. Particularly, the combined occurrence of bacterial pathogen infection and drought may have a great influence on the plant response [31]. There are several common changes in plant responses to drought and pathogens stress: activation of reactive oxygen species scavenging system [32], proline accumulation [33], anthocyanin production [34], lignin deposition [24], reduction of photosynthetic activity [27,35] and alterations in certain other metabolites [23,36].

Goodwin et al. 1988 [37] showed a reduction of stomatal conductance and photosynthesis in symptomatic grapevines infected with Pierce’s disease, which is also a common response of water-limited plants [38]. Since *Xf* causes a blockage of the xylem bringing the plant in water deficit, the transcriptional profile of infected plants should simulate that induced by drought stress. In order to validate this hypothesis, we report data relative to expression of genes strongly related to these stresses in two *O. europaea cv* affected by *Xf*, water deficit, or the combination of *Xf* infection and water deficit, representing de facto, the starting point for further investigations on this topic in the olive tree.

Maintenance of water homeostasis is necessary for various biochemical and physiological processes. RWC is considered an essential indicator of water status in plants, representing the balance between water supply and transpiration rate in leaf tissue [39] and is a meaningful determinant of the drought tolerance of plants. In this regard, Cellina *cultivar* maintains a better water status than that of Leccino *cultivar*, critical for its physiological functioning and survival under drought. A further decrease in RWC in plants with low tolerance against drought (Leccino) was not observed when subjected to pathogen infection or combined stress, while, in the same conditions, was observed a remarkable decrease in Cellina. In addition, mechanisms producing a synergistic effect between water deficit and infection in trees have been introduced in a study of Dutch elm disease (DED) in *Ulmus minor* [40]. The severity of symptoms of DED associated with water stress was increased in plants with large vessels more incline to cavitation. Vessel cavitation is considered a determinant process of the wilting of the plant in stress conditions [41]. Again, as reported by Sabella et al. (2018) [24], *Xf* resistance of olive trees *cv* Leccino could be influenced by lignin amount in the xylem vessels that limit the bacteria movement and the host invasion by slowing down the disease progression.

The different performance of the two *cultivars* to individual stress and combined stress also displays in the different gene expression. According to the literature [42], the accumulation of *DREB* and *DHN* in plants is associated with drought stress tolerance. In fact, the expression level of these genes in vegetative tissues was generally been found to be higher in drought-tolerant *cv* Cellina than in susceptible *cv* Leccino. Other research groups have shown that raising levels of *DREB* expression increase the expression of downstream target genes encoding late embryogenesis abundant (LEA) proteins, also known as dehydrins (DHNs) [43]. Moreover, both genes were overexpressed also in the presence of *Xf*, probably because the bacterium is not recognized by the plant as biotic stress, but rather as abiotic stress related to drought and dehydration allowing the fortification and water loss prevention [44,45]. Water channel proteins are known as aquaporins (AQPs) regulate the movement of water and other small molecules across plant vacuolar and plasma membranes; they are associated with plant tolerance of biotic and abiotic stresses. Different responses of AQPs to water deficit stress were found in drought-resistant and drought-sensitive olive *cultivars* [46]. In our results, mRNA levels in leaves were significantly up-regulated in Cellina, but their expression was lower in Leccino. According to literature, our data indicate that unchanged or down-regulated of aquaporins by water stress may result in reduced cell water permeability and may promote cellular water conservation, demonstrate consequently higher Leccino’s resistance to stresses.

One of the inevitable effects of water deficit, caused by abiotic or biotic factors or by a combination of both, is enhanced ROS production in the chloroplasts, the peroxisomes, and the mitochondria, leading to the abnormalities at the cellular level [47]. However, plants are able to deal with such stressful conditions through increased synthesis of metabolites, including proline, and antioxidant enzymes [47].

In the present study, the increase in the activities of APX, CAT, and SOD as well as of proline content in Cellina and Leccino *cvs* due to stress conditions were observed (Figure 2; Table 1). The higher levels of APX, CAT, and SOD activities shown in Leccino *cv* compared to Cellina underline the effectiveness of Leccino’s antioxidative enzyme system at protecting the cellular apparatus under individual and combined stress conditions. Furthermore, the higher proline accumulation observed in Leccino olive tree under stresses was accompanied by higher activities of SOD, APX and CAT, suggesting that proline accumulation could activate the antioxidative defense mechanism in Leccino *cv* as has been suggested by Ahmed et al. (2009) [48] observing intra-specific differences in the water-stressed olive *cultivars*.

Abiotic and biotic stresses lead to ROS formation and the induction of genes that codify for antioxidant enzymes.

The implication of those genes in furthering plant responses to unfavourable conditions has been well determined in many studies [49,50]. It has been reported that the overexpression of genes encoding antioxidant enzymes origin major tolerance to stress factors in *Arabidopsis thaliana* [51] and rice [52,53]. Moreover, some data indicate that retaining stable gene expression can confer drought tolerance in plants [54,55]. In particular, recently it was evidenced in *O. europaea* that high concentrations of ROS switch on plant defense signalling pathways in fighting *X. fastidiosa* infection [56]. This prompted us to examine the role of oxidative stress genes in order to understand the shared mechanism between individual and combined stresses in *cultivars* considered. As reported by recent studies [27,35] a good performance of photosynthetic apparatus under drought stress is very important also for biotic tolerance of plants, because of influence the antioxidant defense system activity. In particular, we investigated *APX*, *CAT*, *Cu/Zn SOD*, and *Mn SOD* genes, commonly belonging to the oxidative stress scavenging system. As reported in Figure 4 the genes in both individual and combined stresses were induced with similar expression patterns within each *cultivar*. However, a differential expression pattern was observed in the *Xf*-resistant *cultivar* Leccino, which shows a higher expression level compared to the susceptible *cultivar* Cellina. This result appears even more interesting if we consider the lower bacterial content present in Leccino compared to Cellina, suggesting that resistance to *Xf* in Leccino is closely related to the higher activity of these genes, regardless of the infection level.

According to Lamb and Dixon (1997) [57], our findings indicate that the susceptibility to drought stress of Leccino provokes an important accumulation of ROS which acts as a secondary messenger in signal transduction and triggers a higher defense response against the pathogen. In fact, also the defense-associated genes were up-regulated in Leccino infected by *Xf* and, as reported by Giampietruzzi et al. (2016) [58], in a special way for the *LRR-RLK* gene. Also, in our observation, the *PR* and *LRR-RLK* genes were not further induced by the additional stress caused by water deficit [17], suggesting that genes can respond to simultaneous stress in a different way and not always in an additive way, as widely reported in the literature [12,32].

## 4. Materials and Methods

### 4.1. Field Conditions and Plant Material

Trials were carried out in summer on *O. europaea* L. plants, *cvs* Cellina and Leccino, in productive orchards located in Lecce (Apulia, Southern Italy). Selected plants had previously received the same agronomic practices (with differences only in water management, see following paragraphs) and insect control over 3 years, and phytosanitary treatments had been carried out by the farmers according to EU Decision 2015/789.

We used an experimental design with 24 olive trees, 12 *cvs* Cellina and 12 Leccino with an age ranging from 25–35 years. The trials were carried out on sandy soils (76.0% sand, 19.1% silt, 4.9% clay, 1.9% organic matter). The experimental design included four plant conditions: *Xf*-positive trees naturally infected and irrigated (*X. fastidiosa*, three plants/*cultivar*), *Xf*-negative trees and subjected to water deficit (Drought, three plants/*cultivar*), *Xf*-positive trees subjected to water deficit (Combined, three plants/*cultivar*), *Xf*-negative trees and irrigated (Control, 3 plants/*cultivar*).

Samples were collected in summer after four weeks of lack of rainfall. For the irrigated plants the water management has predicted schedule irrigation using the water budget approach according to Marra et al., 2016 [59]. In the month before to the sampling, to the well-irrigated thesis of the plants, 300 l/tree of water were dispensed.

The *Xf*-positive or *Xf*-negative plants were assessed by real-time PCR (qPCR) [60]. All presumed *Xf*-naturally infected or *Xf*-non infected plants were singularly tested each year in the 2016–2018 period. The plants were considered healthy when leaf samples were negative to the *Xf* assay (2016–2018 period). With regards to infected plants, the Cellina and Leccino trees were positive to *Xf* assay since the 2016 test, showing *Ct* values respectively of 24–27 and 28–30. The plants selected were monitored for symptoms caused by natural infection of *Spilocaea oleagina* and *Pseudomonas savastanoi* pv. *savastanoi* during the 12 months before sampling. According to Nicolì et al., 2019 [36], the presence of symptoms was scored using a severity scale (0 = symptomless, 1 = symptoms on few branches (≤ 5), 2 = symptoms on several branches (> 5), and 3 = symptoms uniformly distributed throughout the canopy). In addition, diagnostic tests (real-time PCR) were carried out according to the literature for *Botryosphaeria dothidea* [61], *Colletotrichum* spp., *C. acutatum* and *C. gloeosporioide* [62], *Diplodia seriata* [63], *Phaeomoniella chlamydospore* [64], *Phaeoacremonium aleophilum* and *P. parasiticum* [65,66], *Phytophthora* spp. [67], *Verticillium dahlia* [68].

In order to analyze homogeneous trees, both *Xf-*positive and negative plants were selected according to lower severity (= 1) for *Pseudomonas savastanoi* pv. *savastanoi* and *Spilocaea oleagina* and negative to every other diagnostic test but for *Xf*.

### 4.2. Relative Water Content Measurement

Relative water content (RWC) was carried out following the procedure proposed by Barrs and Weatherley (1962) [69] on fully expanded leaves of similar age, divided into blocks of ten leaves each per treatment. Leaves were excised, weighed fresh (FW) and placed in distilled water in the dark for 24 h to rehydrate. The turgid leaf weight (TW) was measured and then leaves were dried at 80 °C for 48 h and dry weight (DW) was determined. The RWC was calculated as:

RWC = [(FW − DW)/(TW − DW)] × 100

### 4.3. Free Proline Determination

Approximately 0.5 g of powder plant material from control and stressed plants was homogenized in 3% aqueous sulfosalicylic acid. Free proline content was determined according to Bates et al. (1973) [70]. Proline concentration was calculated using L-proline for the standard curve and reported as µmol g FW^−1^.

### 4.4. Antioxidant Enzymes Determinations

Olive leaves (0.5 g) were ground with pestle and mortar in liquid nitrogen. Then, the powder transferred into precooled tubes and 1 mL of 50 mM potassium phosphate buffer (pH 7.0), containing 1 mM EDTA and 4% PVPP was subsequently added to tubes. The homogenate was centrifuged at 15,000 g for 20 min at 4 °C.

The supernatants were collected and used for assays of enzymatic activities. The ascorbate peroxidase (APX, EC1.11.1.11), catalase (CAT, EC 1.11.1.6) and superoxide dismutase (SOD, EC 1.15.1.1) activities were determined according to the method of Giannopolitis and Ries (1977) [71], Chance and Maehly, (1995) [72], and Nakano and Asada (1981) [73], respectively. The values of enzyme activities were expressed as units per mg^−1^ dry weight.

### 4.5. Total RNA Isolation, cDNA Synthesis, and Real-Time PCR Analysis

Total RNA was extracted from leaf samples using TRIzol^®^ (Promega) according to the protocol of the manufacturer. RNA samples were deal with DNase I (Promega) before that their absorbance was read at 260 and 280 nm to define RNA concentration and purity. cDNA synthesis was performed using TaqMan^®^ Reverse Transcription Reagents (Applied Biosystems, Foster City, USA) according to the manufacturer’s instruction, with oligo (dT) 18 as a primer. The RT- PCR was carried out using SYBR Green fluorescent detection in a Real-Time PCR thermal cycler (ABI PRISM 7900 Sequence Detection System, Applied Biosystems, Foster City, CA, USA). The PCR program was: 2 min at 50 °C and 10 min at 95 °C, followed by 45 cycles of 95 °C for 15 s and 60 °C for 1 min. Melting curve analysis was performed after PCR to evaluate the presence of non-specific PCR products and primer dimers. Three biological and three technical replicates were analyzed. The used primers were retrieved from the literature or designed with the software Primer Express Software 3.0 on the mRNA sequences deposited in GenBank. The primers were designed on the genes related to oxidative stress such as superoxide dismutase (*Cu/Zn SOD* and *MnSOD*), catalase (*CAT*), ascorbate peroxidase (*APX*). The genes related to pathogen stress, such as leucine rich repeats-receptors like kinase (*LRR-RLK*) [58] and pathogenesis-related protein 1-like (*PR*). The genes related to drought responses such as aquaporin (*PIP2.1*) [44], dehydration responsive element binding (*DREB*) and dehydrin (*DHN*). To standardize the results the relative abundance of ubiquitin gene (*UBQ*) was used as the internal standard (Table 2). Relative gene expression levels were calculated with the log_2_ 2^−ΔΔCt^ method [74,75]. The efficiency of the target amplification was evaluated for each primer pairs and the corresponding value was used to calculate the fold changes (FC) with the following formula: FC = (1 + E) ^−ΔΔCt^, where ΔΔCt = (Ct_target_ − Ct_UBQ_)_Treatment_ − (Ct_target_ − Ct_UBQ_)_Control_.

### 4.6. Statistical Analysis

All data were reported as the mean ± SD with at least three replications for each leaf olive sample. A two-way ANOVA with the replicates of each measure was carried out on RWC data using *cultivar* and stress conditions as main factors. The data related to gene expression level for each stress (individual and combined stresses) were subjected to one-way ANOVA analysis, followed by Tukey-HSD (honestly significant difference) post hoc test (*p* < 0.05). Also, the statistical analysis was performing using multiple t-tests (FDR = 5%) to under light the differences between *cultivars* for each gene and for each stress. Statistical analyses were performed using GraphPad software, version 6.01.

## 5. Conclusions

The expertise of how abiotic environmental factors influence plant resistance to pathogens and how systems specifically elaborate the response to combined abiotic and biotic stress for disease management is important to breeding programs aimed at improving *Xf* resistance in *O. europaea cultivars* also in the forecast of future climate changes. In this work, we hypothesize that the resistance of Leccino to *Xf* could be related to its lower resistance to water stress, that could lead to the activation of alternative defense pathways which support the plant in *Xf* response, as widely discussed by Ramegowda et al., 2013 [18] about drought-pathogen stress interactions in plants. Due to the critical status of *Xf* epidemy in Salento, here we report urgently the first evidence about host’s transcriptional responses of drought-related genes to natural infection according to water management practices commonly carried out in the area. However, the transcription of further genes, the correlation of gene expression with enzymatic activities and trials with artificially inoculated plants submitted to different levels of water deficit grown in controlled environment should drive further research to confirm this evidence and improve the understanding of the molecular basis of stress resistance.

## Figures and Tables

**Table 1:** Enzyme activities of ascorbate peroxidase (APX), catalase (CAT), and superoxide dismutase (SOD) determined on Cellina di Nardò and Leccino leaves subjected to individual and combined stresses (drought and *Xylella fastidiosa*). Data presented are the means ± SEs (*n=* 5). Within rows, means followed by different low case letters indicate a significant difference (*p* ≤ 0.05) among the *cultivars*, within columns, means followed by different capital letters indicate a significant difference (*p* ≤ 0.05) among the treatments.

	*Enzyme activity (EU mg¯¹ protein)*					
	APX		CAT		SOD	
*Plant* *conditions*	Cellina di Nardò	Leccino	Cellina di Nardò	Leccino	Cellina di Nardò	Leccino
**Control**	3.20 ± 0.03 **b**, **D**	3.66 ± 0.12 **a**, **C**	4.82 ± 0.23 **b**, **C**	5.23 ± 0.10 **a**, C	12.94 ± 0.14 **a**, **D**	13.46 ± 0.42 **a**, **C**
**Drought**	4.34 ± 0.17 **b**, **C**	9.23 ± 0.03 **a**, **B**	8.53 ± 0.43 **b**, **A**	10.73 ± 0.22 **a**, **B**	17.64 ± 0.57 **b**, **B**	27.02 ± 0.99 **a**, **B**
***X.fastidiosa***	7.22 ± 0.07 **b**, **A**	9.27 ± 0.12 **a**, **B**	6.53 ± 0.32 **b**, **B**	15.24 ± 0.39 **a**, **A**	14.08 ± 0.20 **b**, **C**	26.52 ± 0.17 **a**, **B**
**Combined**	4.66 ± 0.10 **b**, **B**	12.30 ± 0.13 **a**, **A**	9.10 ± 0.19 **b**, **A**	11.03 ± 0.03 **a**, **B**	19.33 ± 0.24 **b**, **A**	28.49 ± 0.25 **a**, **A**

**Table 2:** Primers used to evaluate the expression of genes involved in drought stress, stress induced by *Xylella fastidiosa* and combined stresses in *Olea europaea* L. Cellina di Nardò and Leccino.

Functional Categories and Genes	Abbr.	Primer	Sequence 5′-3′	GeneBank
*ROS scavenging activity genes related*				
Ascorbate Peroxidase	APX	*Oe* *APXF*	CAAAAACTGCGCCCCTATAA	XM023040324.1
		*Oe* *APXR*	ACAGCAACAACACCAGCAAG	
Catalase	CAT	*Oe* *CATF*	GGATCCAGCCAGACAAGAGA	JQ429793
		*Oe* *CATR*	TTGGCCTTACATTGAGACGA	
Manganese Superoxide Dismutase	MnSOD	*Oe* *MnSODF*	CTCCTGTTCGTGAAGGTGGT	AF427107
		*Oe* *MnSODR*	GTGTCCAGACCAAGCCAAAT	
Copper/Zinc Superoxide Dismutase	Cu/ZnSOD	*OeCu/ZnSODF*	CCATGCTGGTGATCTTGGTA	AF191342
		*OeCu/ZnSODR*	CAGTTCATGACCACCCCTTC	
*Pathogen genes related*				
Leucine Rich Repeats	LRR-RLK	*OeLRRF*	CAACACAAGGCTTTTGGGACTT	XP_006367556.1 *
		*Oe* *LRRR*	TGTCATTGGTGCTTGTTGGT	
Pathogenesis Protein Related	PR	*OePRF*	AACAAGGCTCGTGCAGAAGT	XM023013713
		*OePRR*	TCGACCCATGATCATAGCAA	
*Water deficit genes related*				
Aquaporine	PIP2.1	*OePIP2.1F* *OePIP2.1R*	TCTCGGGCCCTTGTTTTAGA AAAGAGAGGCCAGCAACCG	DQ202709
Dehydration responsive element binding	DREB	*OeDREBF* *OeDREBR*	ACATGTTCTCCGCTCAGCTT GTGCCTCGTCTCCTTGAAAA	EF635424.1
Dehydrin	DHN	*Oe* *DHNF*	GGTTTGAAGGGGAAGGTTTC	KR349290.1
		*Oe* *DHNR*	CTTCCTCAGCCTTCTTGTGG	
*Reference gene*				
Polyubiquitin	UBQ	*OeUBQF*	GGTGGCCTCTAAATGTTCTTCTACTG	AF429430 *
		*OeUBQR*	CACACAGACTTCATTAGAAAGACAATCA	

* LRR-RLK and UBQ primers were retrieved from Giampietruzzi et al., 2016 [58].

## References

[1] Abuqamar S., Luo H., Laluk K., Mickelbart M.V., Mengiste T. (2009). Crosstalk between biotic and abiotic stress responses in tomato is mediated by the AIM1 transcription factor. Plant J..

[2] Mengiste T., Chen X., Salmeron J., Dietrich R. (2003). The *Botrytis susceptible1* gene encodes an R2R3MYB transcription factor protein that is required for biotic and abiotic stress responses in *Arabidopsis*. Plant Cell.

[3] Suzuki N., Rizhsky L., Liang H., Shuman J., Shulaev V., Mittler R. (2005). Enhanced tolerance to environmental stress in transgenic plants expressing the transcriptional coactivator multiprotein bridging factor 1c. Plant Physiol..

[4] Narsai R., Wang C., Chen J., Wu J., Shou H., Whelan J. (2013). Antagonistic, overlapping and distinct responses to biotic stress in rice (*Oryza sativa*) and interactions with abiotic stress. BMC Genomics..

[5] Shaik R., Ramakrishna W. (2013). Genes and co-expression modules common to drought and bacterial stress responses in *Arabidopsis* and rice. PLoS ONE.

[6] Shaik R., Ramakrishna W. (2014). Machine learning approaches distinguish multiple stress conditions using stress-responsive genes and identify candidate genes for broad resistance in rice. Plant Physiol..

[7] Sharma R., Vleesschauwer D.D., Sharma M.K., Ronald P.C. (2013). Recent advances in dissecting stress-regulatory crosstalk in rice. Mol. Plant.

[8] Atkinson N.J., Lilley C.J., Urwin P.E. (2013). Identification of genes involved in the response of *Arabidopsis* to simultaneous biotic and abiotic stresses. Plant Physiol..

[9] Bostock R.M., Pye M.F., Roubtsova T.V. (2014). Predisposition in plant disease: Exploiting the nexus in abiotic and biotic stress perception and response. Annu. Rev. Phytopathol..

[10] Kissoudis C., Van de Wiel C., Visser R.G.F., Van Der Linden G. (2014). Enhancing crop resilience to combined abiotic and biotic stress through the dissection of physiological and molecular crosstalk. Front. Plant Sci..

[11] Mittler R. (2006). Abiotic stress, the field environment and stress combination. Trends Plant Sci..

[12] Pandey P., Ramegowda V., Senthil-Kumar M. (2015). Shared and unique responses of plants to multiple individual stresses and stress combinations: Physiological and molecular mechanisms. Front. Plant Sci..

[13] Prasch C.M., Sonnewald U. (2015). Signaling events in plants: Stress factors in combination change the picture. Environ. Exp. Bot..

[14] Prasch C.M., Sonnewald U. (2013). Simultaneous application of heat, drought, and virus to *Arabidopsis* plants reveals significant shifts in signaling networks. Plant Physiol..

[15] Rasmussen S., Barah P., Suarez-Rodriguez M.C., Bressendorf S., Friis P., Costantino P. (2013). Transcriptome responses to combinations of stresses in *Arabidopsis*. Plant Physiol..

[16] Mattson W.J., Haack R.A., Barbosa P., Schultz J.C. (1987). The role of drought stress in provoking outbreaks of phytophagous insects. Insect Outbreaks.

[17] Choi H.K., Iandolino A., Silva F.G., Cook D.R. (2013). Water deficit modulates the response of *Vitis vinifera* to the Pierce’s disease pathogen *Xylella fastidiosa*. Mol. Plant-Microbe Interact..

[18] Ramegowda V., Senthil-Kumar M., Ishiga Y., Kaundal A., Udayakumar M., Mysore K.S. (2013). Drought stress acclimation imparts tolerance to *Sclerotinia sclerotirum* and *Pseudomonas syringae* in *Nicotiana benthamiana*. Int. J. Mol. Sci..

[19] Gupta A., Sarkar A.K., Senthil-Kumar M. (2016). Global transcriptional analysis reveals unique and shared response in *Arabidopsis thaliana* exposed to combined drought and pathogen stress. Front. Plant Sci..

[20] Mittler R., Vanderauwera S., Gollery M., Van Breusegem F. (2004). The reactive oxygen gene network in plants. Trends Plant Sci..

[21] Chiappetta A., Muto A., Bruno L., Woloszynska M., Vanlijsebettens M., Bitonti M.B. (2015). A dehydrin gene isolated from feral olive enhances drought tolerance in *Arabidopsis* transgenic plants. Front. Plant Sci..

[22] Saponari M., Boscia D., Nigro F., Martelli G.P. (2013). Identification of DNA sequences related to *Xylella fastidiosa* in oleander, almond and olive trees exhibiting leaf scorch symptoms in Apulia (southern Italy). J. Plant Pathol..

[23] Luvisi A., Aprile A., Sabella E., Vergine M., Nicolì F., Nutricati E., Miceli A., Negro C., De Bellis L. (2017). *Xylella fastidiosa* subsp. *pauca* (CoDiRO strain) infection in four olive (*Olea europaea* L.) *cultivars*: Profile of phenolic compounds in leaves and progression of leaf scorch symptoms. Phytopathol. Mediterr..

[24] Sabella E., Luvisi A., Aprile A., Negro C., Vergine M., Nicolì F., Miceli A., De Bellis L. (2018). *Xylella fastidiosa* induces differential expression of lignification related-genes and lignin accumulation in tolerant olive trees *cv*. Leccino. J. Plant Physiol..

[25] Loconsole G., Saponari M., Boscia D., D’Attoma G., Morelli M., Martelli G., Almeida R.P.P. (2016). Intercepted isolates of *Xylella fastidiosa* in Europe reveal novel genetic diversity. Eur. J. Plant Pathol..

[26] Cardinale M., Luvisi A., Meyer J.B., Sabella E., De Bellis L., Cruz A.C., Ampatzidis Y., Cherubini P. (2018). Specific Fluorescence in Situ Hybridization (FISH) Test to Highlight Colonization of Xylem Vessels by *Xylella fastidiosa* in Naturally Infected Olive Trees (*Olea europaea* L.). Front. Plant Sci..

[27] Dąbrowski P., Baczewska-Dąbrowska A.H., Kalaji H.M., Goltsev V., Paunov M., Rapacz M., Wójcik-Jagła M., Pawluśkiewicz B., Bąba W., Brestic M. (2019). Exploration of chlorophyll a fluorescence and plant gas exchange parameters as indicators of drought tolerance in perennial ryegrass. Sensors.

[28] Bosso L., Febbraro M., Cristinzio G., Zoina A., Russo D. (2016). Shedding light on the effects of climate change on the potential distribution of *Xylella fastidiosa*. Biol. Invasions.

[29] Keller R.P., Lodge D.M., Finnoff D.C. (2007). Risk assessment for invasive species produces net bioeconomic benefits. Proc. Natl. Acad. Sci. USA.

[30] IPCC (2013). Principles Governing IPCC Work. https://www.ipcc.ch/site/assets/uploads/sites/2/2019/05/SR15_Chapter1_Low_Res.pdf.

[31] Ramegowda V., Senthil-Kumar M., Udayakumar M., Kirankumar S.M. (2013). A high-throughput virus induced gene silencing protocol identifies genes involved in multistress tolerance. BMC Plant Biol..

[32] Choudhury F.K., Rivero R.M., Blumwald E., Mittler R. (2017). Reactive oxygen species, abiotic stress and stress combination. Plant J..

[33] Zarattini M., Forlani G. (2017). Toward unveiling the mechanisms for transcriptional regulation of proline biosynthesis in the plant cell response to biotic and abiotic stress conditions. Front. Plant Sci..

[34] Cai Z., Riedel H., Saw N.M.M.T., Kütük O., Mewis I., Jäger H., Knorr D., Smetanska I. (2011). Effects of pulsed electric field on secondary metabolism of *Vitis vinifera* L. *cv*. Gamay Fréaux suspension culture and exudates. Appl. Biochem. Biotechnol..

[35] Mao H., Chen M., Su Y., Wu N., Yuan M., Yuan S., Brestic M., Zivcak M., Zhang H., Chen Y. (2018). Comparison on photosynthesis and antioxidant defense systems in wheat with different ploidy levels and octoploid triticale. Int. J. Mol. Sci..

[36] Nicolì F., Negro C., Nutricati E., Vergine M., Aprile A., Sabella E., Damiano G., De Bellis L., Luvisi A. (2018). Accumulation of azelaic acid in *Xylella fastidiosa*-infected olive trees: A mobile metabolite for health screening. Phytopathology.

[37] Goodwin P.H., DeVay J.E., Meredith C.P. (1988). Roles of water stress and phytotoxins in the development of Pierce’s disease of the grapevine. Physiol. Mol. Plant Pathol..

[38] Hsiao T.C. (1973). Plant responses to water stress. Annu. Rev. Plant Physiol..

[39] Lugojan C., Ciulca S. (2011). Evaluation of relative water content in winter wheat. J. Hortic. For. Biotechnol..

[40] Solla A., Gil L. (2002). Influence of water stress on Dutch elm disease symptoms in *Ulmus minor*. Can. J. Bot..

[41] Sabella E., Aprile A., Genga A., Siciliano T., Nutricati E., Nicolì F., Vergine M., Negro C., De Bellis L., Luvisi A. (2019). Xylem cavitation susceptibility and refilling mechanisms in olive trees infected by *Xylella fastidiosa*. Sci. Rep..

[42] Hu L., Wang Z., Du H., Huang B. (2010). Differential accumulation of dehydrins in response to water stress for hybrid and common bermudagrass genotypes differing in drought tolerance. J. Plant Physiol..

[43] Battaglia M., Olvera-Carrillo Garciarrubio A., Campos F., Covarrubias A.A. (2008). The enigmatic LEA proteins and other hydrophilins. Plant Physiol..

[44] Secchi F., Lovisolo C., Schubert A. (2007). Expression of *Oe*PIP2.1 aquaporin gene and water relations of *Olea europaea* twigs during drought stress and recovery. Ann. Appl. Biol..

[45] Rapicavoli J.N., Blanco-Ulate B., Muszyński A., Figueroa-Balderas R., Morales-Cruz A., Azadi P., Dobruchowska J.M., Castro C., Cantu D., Roper M.C. (2018). Lipopolysaccharide O-antigen delays plant innate immune recognition of *Xylella fastidiosa*. Nat. Commun..

[46] Roper C., Castro C., Ingel B. (2019). *Xylella fastidiosa*: Bacterial parasitism with hallmarks of commensalism. Curr. Opin. Plant Biol..

[47] Hayat S., Yadav S., Wani A.S., Irfan M., Alyemini M.N., Ahmad A. (2012). Impact of sodium nitroprusside on nitrate reductase, proline and antioxidant system in *Solanum lycopersicum* under salinity stress. Hort. Environ. Biotechnol..

[48] Ben Ahmed C., Ben Rouinab B., Sensoyc S., Boukhrisa M., Ben Abdallah F. (2009). Changes in gas exchange, proline accumulation and antioxidative enzyme activities in three olive *cultivars* under contrasting water availability regimes. Environ. Exp. Bot..

[49] Xu F.J., Jin C.W., Liu W.J., Zhang Y.S., Lin X.Y. (2011). Pretreatment with H_2_O_2_ alleviates aluminum-induced oxidative stress in wheat seedlings. J. Integr. Plant Biol..

[50] Zhang Y., Wang X., Li Y., Wu L., Zhou H., Zhang G., Ma Z. (2013). Ectopic expression of a novel Ser/Thr protein kinase from cotton (*Gossypium barbadense*), enhances resistance to *Verticillium dahliae* infection and oxidative stress in *Arabidopsis*. Plant Cell Rep..

[51] Rossel J.B., Walter P.B., Hendrickson L., Chow W.S., Poole A., Mullineaux P.M., Pogson B.J. (2006). A mutation affecting *Ascorbate peroxidase 2* gene expression reveals a link between responses to high light and drought tolerance. Plant Cell Environ..

[52] Prashanth S.R., Sadhasivam V., Parida A. (2008). Over expression of cytosolic copper/zinc superoxide dismutase from a mangrove plant *Avicennia marina* in indica rice var Pusa Basmati-1 confers abiotic stress tolerance. Transgenic Res..

[53] Badawi G.H., Kawano N., Yamauchi Y., Shimada E., Sasaki R., Kubo A., Tanaka K. (2004). Over-expression of ascorbate peroxidase in tobacco chloroplasts enhances the tolerance to salt stress and water deficit. Physiol. Plant..

[54] Bian S., Jiang Y. (2009). Reactive oxygen species, antioxidant enzyme activities and gene expression patterns in leaves and roots of Kentucky bluegrass in response to drought stress and recovery. Sci. Hort..

[55] Jiang H.W., Liu M.J., Chen I.C., Huang C.H., Chao L.Y., Hsieh H.L. (2010). A glutathione S-transferase regulated by light and hormones participates in the modulation of *Arabidopsis* seedling development. Plant Physiol..

[56] Novelli S., Gismondi A., Di Marco G., Canuti L., Nanni V., Caninelli A. (2019). Plant defense factors involved in *Olea europaea* resistance against *Xylella fastidiosa* infection. J. Plant Res..

[57] Lamb C., Dixon R. (1997). The oxidative burst in plant disease resistance. Annu. Rev. Plant. Physiol. Plant Mol. Biol..

[58] Giampetruzzi A., Morelli M., Saponari M., Loconsole G., Chiumenti M., Boscia D., Savino V.N., Martelli G.P., Saldarelli P. (2016). Transcriptome profiling of two olive *cultivars* in response to infection by the CoDiRO strain of *Xylella fastidiosa* subsp. pauca. BMC Genomics.

[59] Marra F.P., Marino G., Marchese A., Caruso T. (2016). Effects of different irrigation regimes on a super-high-density olive grove *cv*. “Arbequina”: Vegetative growth, productivity and polyphenol content of the oil. Irrig. Sci..

[60] Harper S.J., Ward L.I., Clover G.R.G. (2010). Development of LAMP and Real-Time PCR Methods for the rapid detection of *Xylella fastidiosa* for quarantine and field applications. Phytopatology.

[61] Romanazzi G., Murolo S., Pizzichini L., Nardi S. (2009). Esca in young and mature vineyards: And molecular diagnosis of the associated fungi. Eur. J. Plant Pathol..

[62] Garrido C., Carbú M., Fernández-Acero F.J., Boonha N., Colyer A., Cantoral J.M., Budge G. (2009). Development of protocols for detection of *Colletotrichum acutatum* and monitoring of strawberry anthracnose using qPCR. Plant Pathol..

[63] Martín M.T., Cuesta M.J., Martín L. (2014). Development of SCAR primers for PCR assay to detect *Diplodia seriata*. Int. Sch. Res. Not..

[64] Martín M.T., Cobos R., Martín L., López-Enríquez L. (2012). qPCR Detection of *Phaeomoniella chlamydospora* and *Phaeoacremonium aleophilum*. Appl. Environ. Microb..

[65] Aroca A., Raposo R., Lunello P. (2008). A biomarker for the identification of four *Phaeoacremonium* species using the *β-tubulin* gene as the target sequence. Appl. Microbiol. Biot..

[66] Carlucci A., Lops F., Marchi G., Mugnai L., Surico G. (2013). Has *Xylella fastidiosa* “Chosen” Olive Trees to Establish in the Mediterranean Basin?. Phytopathol. Mediterr..

[67] Drenth A., Wagels G., Smith B., Sendall B., O’Dwyer C., Irvine G., Irwin J.A.G. (2006). Development of a DNA-based method for detection and identification of *Phytophthora* species. Australas. Plant. Path..

[68] Bilodeau G.J., Koike S.T., Uribe P., Martin F.N. (2012). Development of an assay for rapid detection and quantification of *Verticillium dahliae* in soil. Phytopathology.

[69] Barrs H.D., Weatherley P.E. (1962). A re-examination of the relative turgidity technique for estimanting water deficits in leaves. Austral. J. Biol. Sci..

[70] Bates L.S., Waldren R.P., Teare I.D. (1973). Rapid determination of free proline for water-stress studies. Plant Soil..

[71] Giannopolitis C.N., Rise S.K. (1977). Superoxide dismutases. I. Occurrence in higher plants. Plant Physiol..

[72] Nakano Y., Asada K. (1980). Spinach chloroplasts scavenge hydrogenperoxide on illumination. Plant Cell Physiol..

[73] Chance B., Maehly S.K. (1955). Assay of catalase and peroxidase. Methods Enzymol..

[74] Livak K.J., Schmittgen T.D. (2001). Analysis of relative gene expression data using real-time quantitative PCR and the 2^− ΔΔCT^ method. Methods.

[75] Quackenbush J. (2002). Microarray data normalization and transformation. Nat. Genet..

